# High-dose drug heat map analysis for drug safety and efficacy in multi-spheroid brain normal cells and GBM patient-derived cells

**DOI:** 10.1371/journal.pone.0251998

**Published:** 2021-12-02

**Authors:** Sang-Yun Lee, Yvonne Teng, Miseol Son, Bosung Ku, Ho Sang Moon, Vinay Tergaonkar, Pierce Kah-Hoe Chow, Dong Woo Lee, Do-Hyun Nam

**Affiliations:** 1 Department of Health Sciences and Technology, SAIHST, Sungkyunkwan University, Seoul, Korea; 2 Central R & D Center, Medical & Bio Device (MBD) Co., Ltd, Suwon, Republic of Korea; 3 Research & Development Department, AVATAMED Pte. Ltd., Singapore, Singapore; 4 Laboratory of NFκB Signaling, Institute of Molecular and Cell Biology (IMCB), A*STAR (Agency for Science, Technology and Research), Singapore, Singapore; 5 Department of Pathology, Yong Loo Lin School of Medicine, National University of Singapore (NUS), Singapore, Singapore; 6 Division of Surgery and Surgical Oncology, National Cancer Centre Singapore (NCCS), Singapore, Singapore; 7 Department of Hepatopancreatobiliary and Transplant Surgery, Singapore General Hospital (SGH), Singapore, Singapore; 8 Surgery Academic Clinical Programme, Duke-NUS Medical School, Singapore, Singapore; 9 Faculty (Senior Group Leader), Genome Institute of Singapore (GIS), Singapore, Singapore; 10 Research Director, Institute of Molecular Cell Biology (IMCB), Singapore, Singapore; 11 Department of Biomedical Engineering, Konyang University, Daejon, Korea; 12 Department of Neurosurgery, Samsung Medical Center, Sungkyunkwan University School of Medicine, Seoul, Korea; Lobachevsky University, RUSSIAN FEDERATION

## Abstract

To test the safety and efficacy of drugs via a high does drug heat map, a multi-spheroids array chip was developed by adopting a micropillar and microwell structure. In the chip, patient-derived cells were encapsulated in alginate and grown to maturity for more than 7 days to form cancer multi-spheroids. Multi-spheroids grown in conventional well plates require many cells and are easily damaged as a result of multiple pipetting during maintenance culture or experimental procedures. To address these issues, we applied a micropillar and microwell structure to the multi-spheroids array. Patient-derived cells from patients with Glioblastoma (GBM), the most common and lethal form of central nervous system cancer, were used to validate the array chip performance. After forming multi-spheroids with a diameter greater than 100μm in a 12×36 pillar array chip (25mm × 75mm), we tested 70 drug compounds (6 replicates) using a high-dose to determine safety and efficacy for drug candidates. Comparing the drug response of multi-spheroids derived from normal cells and cancer cells, we found that four compounds (Dacomitinib, Cediranib, LY2835219, BGJ398) did not show toxicity to astrocyte cell and were efficacious to patient-derived GBM cells.

## Introduction

The 2D monolayer cell culture model has traditionally been used to evaluate the response of cancer cells to different anti-cancer drug compounds. However, when cancer cells are attached to a plastic dish, they grow as a single layer which is morphologically different from the 3D architecture of animal cells grown *in vivo*. The gene expression of monolayer 2D cells also differs from cells grown in a 3D cell culture model [1]. Furthermore, the results of drug screening using a 2D cell culture model are vastly different from those results collected using a 3D culture model [2–6]. Given these differences, there is great interest and motivation by many research groups to improve 3D cell culturing techniques. Generally, 3D cell culture methods can be categorized into scaffold-free methods that allow cells to grow together without an extra-cellular matrix (ECM) and scaffold-dependent methods that cultivate cells with an ECM. Recently, a scaffold-dependent *in vitro* method was developed to form multi-spheroids that recapitulate physiological conditions [7–9]. The resulting multi-spheroids could be used in biomedical research, genomic analysis of various diseases, and therapeutic studies [10–13]. Multi-spheroids could be particularly powerful tools in drug discovery [14, 15] and personalized cancer treatments [9, 16].

**Fig 1:**
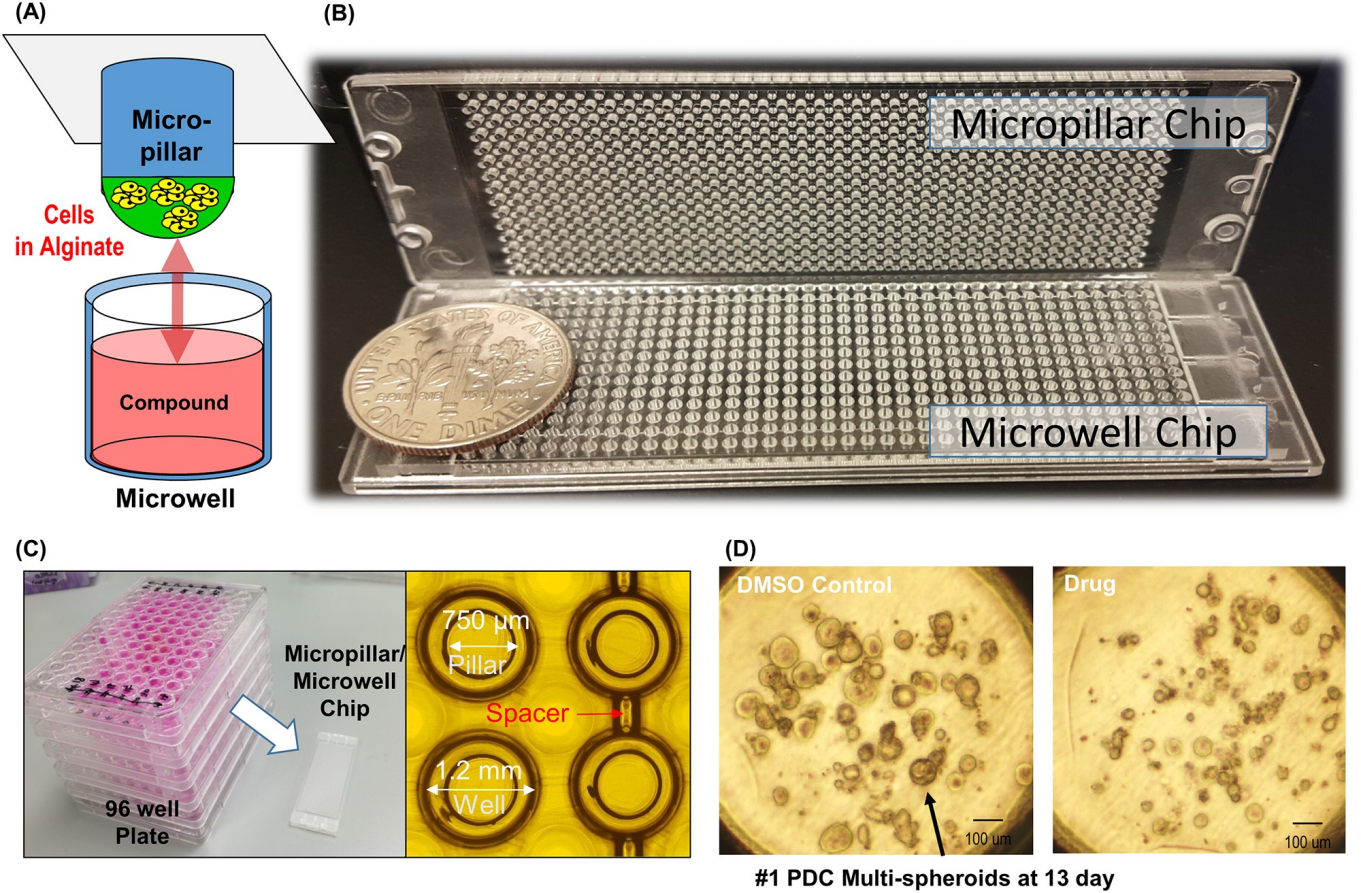
Cancer multi-spheroids array chip designed based on micropillar and microwell chips. (A) the micropillar chip containing human cells in alginate was combined (or “stamped”) with the microwell chip. (B) The fabricated micropillar/microwell chips. (C) photo of the combined micropillar and microwell chip compared with conventional 96 well plate. (D) Representative multi-spheroids images of #PDC1 with DMSO control and drug compound.

Growing multi-spheroids in a high throughput manner is technically difficult due to the potential damage resulting from pipetting and manipulation. To address this issue, we applied a micropillar and microwell structure to the multi-spheroids array as shown in Fig 1A–1C. Previous studies from our lab reported the design of a micropillar and microwell chip for culturing 3D cells for use in drug compound screening tests [17–19]. However, only single cell or small spheroids were exposed to the compounds. In the current study, multi-spheroids arrays were formed on micropillar chips by growing small spheroids to clumps of cells 100 μm-diameter or larger, as shown in spot images in Fig 1D. Patient-derived cells cultured for more than 7 days formed multi-tumor spheroids in colony cells. The spheroid formed in the conventional U-bottom well is a single spheroid. Cells are aggregated in U-bottom without ECM. Unlike the U-bottom well method, the multi-spheroid model encapsulates cells in the ECM and forms several spheroids from each cell in the ECM. While the single spheroid is formed by the aggregation from the cells seeded, the multi-spheroids are formed individually by proliferation from each cell seeded in one ECM spot, creating multiple spheroids. Culturing multi-spheroids in this manner prevented damage to the multi-spheroids when pipetting during cell maintenance as the micropillar chip was transferred directly to a new microwell filled with fresh media. We used the multi-spheroids array to evaluate the efficacy and toxicity of different compounds by exposing brain cells derived from patients with Glioblastoma (GBM), the most common and lethal form of central nervous system cancer to the compounds. Samsung Medical Center Biobank and their genetic profiles well matched with original tissue in the previous our research [20, 21]. After forming more than 100 μm-diameter multi-spheroids in 12 × 36 pillar array chip (25mm × 75mm), we tested 70 drug compounds (6 replicates each) to evaluate individual drug efficacy and toxicity in high dose. With limited number of patient-derived cells (PDC), the purpose of this paper is the primary screening to select efficacy drugs among many drugs without risk of brain normal cell cytotoxicity. Therefore, we choose a high-dose of 20 μM to exclude cytotoxicity compound in high-dose. Under this condition, we selected high efficacy drug candidates without cytotoxicity risk by evaluating drug response of multi-spheroids derived from normal cells (astrocyte) and cancer cells (GBM PDC).

For forming multi-spheroids, alginate hydrogel was used. Alginate lacks adhesive ligands and has limited ability to mimic microenvironments such as microglia and the blood brain barrier. However, alginates of non-animal origin have good mechanical properties and good reliability for use in high-throughput screening [22]. Gelation by ions is temperature-independent, so the gelation of alginate can also be well controlled, which is very good for high-throughput screening.

## Materials and methods

### Experimental procedures

**Fig 2:**
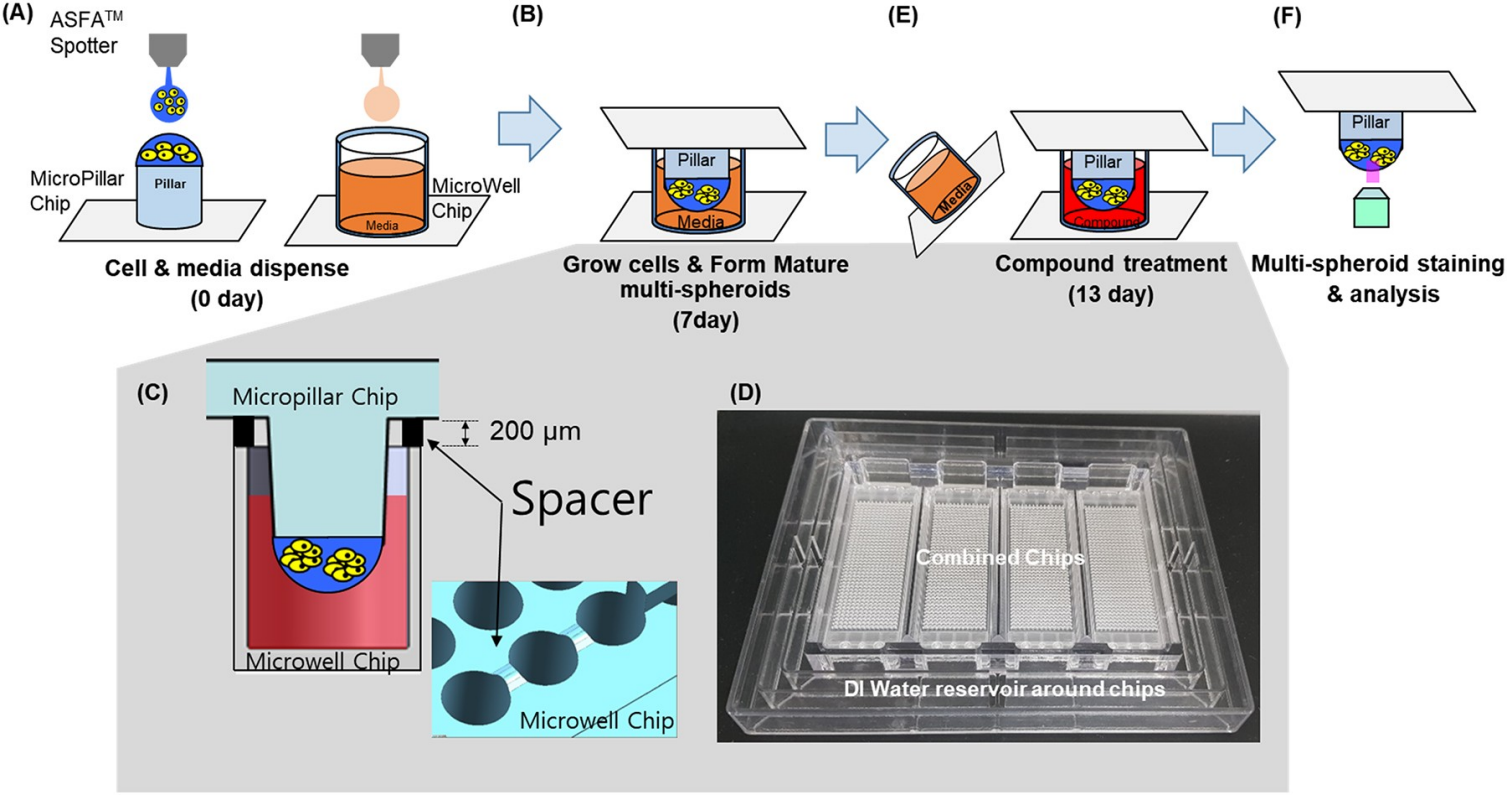
Experimental procedure and fabricated chips. (A) Cell-alginate mixtures and media dispensed on micropillar and in microwell. (B) Cells were immobilized in alginate on the surface of the micropillars and dipped in the microwells containing growth media for 7 days to form mature multi-spheroids. (C) Spacer on the microwell chip created gaps between pillar and well chips for CO_2_ gas exchange during long term cell culture. (D) Chip incubation chambers designed to prevent media evaporation during long term cell culture. (E) Compounds dispensed into the microwells and multi-spheroids are exposed to the compounds by moving the micropillar chip to a new microwell chip. (F) Multi-spheroids are stained with Calcein AM, and the dried alginate spot on the micropillar chip is scanned for data analysis.

For drug analyses, approximately 100 cells patient-derived GBM cells and astrocytes provided by Samsung Medical Center Biobank were used. The genetic profiles of GBM cells were well matched with tissue used in prior research [20, 21]. GBM cells suspended in 50 nL with 0.5% (w/w) alginate were automatically dispensed onto a micropillar chip by using ASFA™ Spotter ST (Medical & Bio Decision, South Korea). The ASFA™ Spotter ST uses a solenoid valve (The Lee Company, USA) for dispensing 50 nL droplets of the cell–alginate mixture and 1 μL of media or compounds. After dispensing the cells and media in micropillar and microwell respectively (Fig 2A), the micropillar chip containing GBM cells in alginate was combined (or “stamped”) with the microwell chip filled with 1 μL the fresh media (Fig 2B). Each microwell chip contained 532 wells, each 1.2 mm in diameter, combined as shown in Fig 2B. Cells were grown for 1 day at 37°C as the cells were dissociated with trypsin to make single cells and needed to be stabilized in the alginate spots before being treated with drugs. After 1 day of incubation, the micropillar chip containing the cells was moved to a new microwell chip filled with the drugs to be tested in the single cell condition. For drug analysis in multi-spheroid conditions, cells dispensed on the micropillar chip are cultured for a period of 7 days. After incubation for 3 days at 37℃, cells started to form spheres. We replaced the media with fresh media every 2–3 days and allowed the cells to continue growing for up to 7 days or when the size of spheres become larger than 100 μm. Micropillar chips were spaced 200 μm from the microwell chip to enable uniform CO_2_ penetration into the multi-spheroids (Fig 2C). An incubation chamber was used while incubating the cells on the chip to prevent media evaporation (Fig 2D). A high-dose drug heat map model that dispenses 20 μM of 70 different drugs into the microwell was designed to evaluate efficacy using a single chip. Using this design, each 70-drug model had six replicates. At day 7, drug compounds were added to spheroids (Fig 2E). In Fig 2F, cell viability was measured with Calcein AM live cell staining dye (4 mM stock from Invitrogen), which stains viable cells with green fluorescence. Due to the low assay volume utilized by the microchip (1 ul) for high throughput screen, the ATP or MTT assay did not generate enough signal to measure cell viability. Alternatively, we selected Calcein AM to measure live cell morphology. Calcein AM stain is a conventional method used in staining 3D spheroids and organoids. Calcein AM fluorescence reagent can freely penetrate into cells and display active cell-specific fluorescence by Glutathione-S-transferase reaction. After the transferase reaction, the Calcein AM reagent changes its structure and does not leak out of the cytoplasm. Prior studies have similarly used drug screening assays with calcein AM live cell staining reagent in 3D cell culture-based drug screening platforms [17–19, 23]. The fabricated micropillar/microwell chips were compared with five 96-well plates in (Fig 1C). A fully grown cancer multi-spheroids with and without addition of drug compounds is shown in Fig 1D.

### Fabrication of micropillar/microwell chips and incubation chamber for multi-spheroids array

Previously, the micropillar/microwell chip platform was used for short term cell culture to form spheroids. When we applied this to long term multi-spheroids cell culture, we found that the low volume of media (1 μL) in each microwell evaporated quickly and there was a lack of CO_2_ supply in the tightly packed chips. To solve these technical issues, the micropillar/microwell chips were modified. When the micropillar and microwell chip were tightly sealed to prevent evaporation, there was insufficient CO_2_ in the microwell. To address this issue, a gap was created between the micropillar and the microwell chips to allow CO_2_ to flow into the wells. The modified micropillar and microwell chips were manufactured by plastic injection molding. The micropillar chip was made of polystyrene-*co*-maleic anhydride (PS-MA) and contained 532 micropillars (0.75 mm pillar diameter and 1.5 mm pillar-to-pillar distance). PS-MA provided a reactive functionality to covalently attach poly-L-lysine (PLL), ultimately attaching alginate spots by ionic interactions. Plastic molding was performed with an injection molder (Sodic Plustech Inc., USA).

The incubation chamber for the micropillar/microwell chips (Fig 2D) was fabricated by cutting cyclic olefin copolymer (COC) with a computer numerical control (CNC) machine. COC was selected because of its high transparency, excellent biocompatibility, and adequate stiffness for physical machining. As shown in Fig 2D, the reservoir around four combined chips was filled with deionized (DI) water to prevent evaporation. It was observed that 5.3% of media evaporated in the incubation chamber during a period of 13 days.

### Astrocyte and patient-derived cell culture

We purchased NHA-astrocyte AGM (LONZA, Cat. No:cc-2565) and cultured it with ABM Basal media (LONZA, Cat. No:cc-3187) that was added with AGM SingleQuot Kit Suppl.& Growth Factors (LONZA, Cat. No:cc-4123). Patient-derived GBM cells were obtained from GBM patients who underwent brain tumor removal surgery at the Samsung Medical Center (Seoul, Korea). Informed consent was obtained from all patients. Tumor specimens were resected from four patients who were diagnosed with glioblastoma. Following a previously reported procedure [13], surgical samples were enzymatically dissociated into single cells. Four patient-derived cells were obtained from four GBM patients. Dissociated GBM cells were cultured in cell-culture flasks (from Eppendorf, T-75) filled with Neurobasal A (NBA) conditioned media. The NBA conditioned media was comprised of N2 and B27 supplements (0.53 each; Invitrogen) and human recombinant bFGF and EGF (25 ng/ml each; R&D Systems), hereafter, referred as NBE condition media. Cell flasks were placed in a humidified 5% CO_2_ incubator (Sheldon Mfg., Inc.) at 37°C. The cells were routinely passed every 4 days at 70% confluence. For the experiment, cell suspensions were collected in a 50-ml falcon tube from the culture flask. GBM cells were then suspended in 5 mL of NBE condition media. After centrifugation at 2000 rpm for 3 min, the supernatant was removed, and the cells were re-suspended with NBA conditioned media to a final concentration of 10 × 10^6^ cells/mL. The number of cells in the NBA conditioned media was calculated with the AccuChip automatic cell counting kit (Digital Bio, Inc). The rest of the cells were seeded at a concentration of 2 × 10^6^ cells in a T-75 flask containing 15 mL of NBA conditioned media. Given that *in vitro* tumor cell expansion can affect biological properties including drug sensitivity, cells were frozen or used within 4 weeks after biopsy, with their cell culture being limited to 10 passages.

### Efficacy/toxicity test

To validate the multi-spheroids array chip, 70 compounds were evaluated using four PDCs and normal astrocytes (Normal brain cell) for drug efficacy and toxicity. Compounds that showed low cytotoxicity against astrocytes and high efficacy against the four PDCs were selected as the best candidates for GBM cancer cell culture. For experimental testing, we selected clinical cancer drugs from current clinical trials and standard target oncology drugs. all drugs are dissolved in DMSO. A total of 70 compounds whose targets are well-known including: epidermal growth factor receptor (EGFR), phosphoinositide 3-kinase (PI3k), mechanistic target of rapamycin (mTOR), vascular endothelial growth factor (VEGFR), c-mesench.ymal-epithelial transition factor (c-Met), and fibroblast growth factor receptors (FGFR) were selected. These compounds are in phase III or phase IV trials or are oncology drugs approved by the US Food and Drug Administration (FDA). Due to the limited number of patient-derived cells, the purpose of this paper was to conduct a primary screening to select high-efficacy drugs without risk of brain cell cytotoxicity. Therefore, we choose a high dose of 20 μM of TMZ, one of the most used drugs for GBM [24].

**Fig 3:**
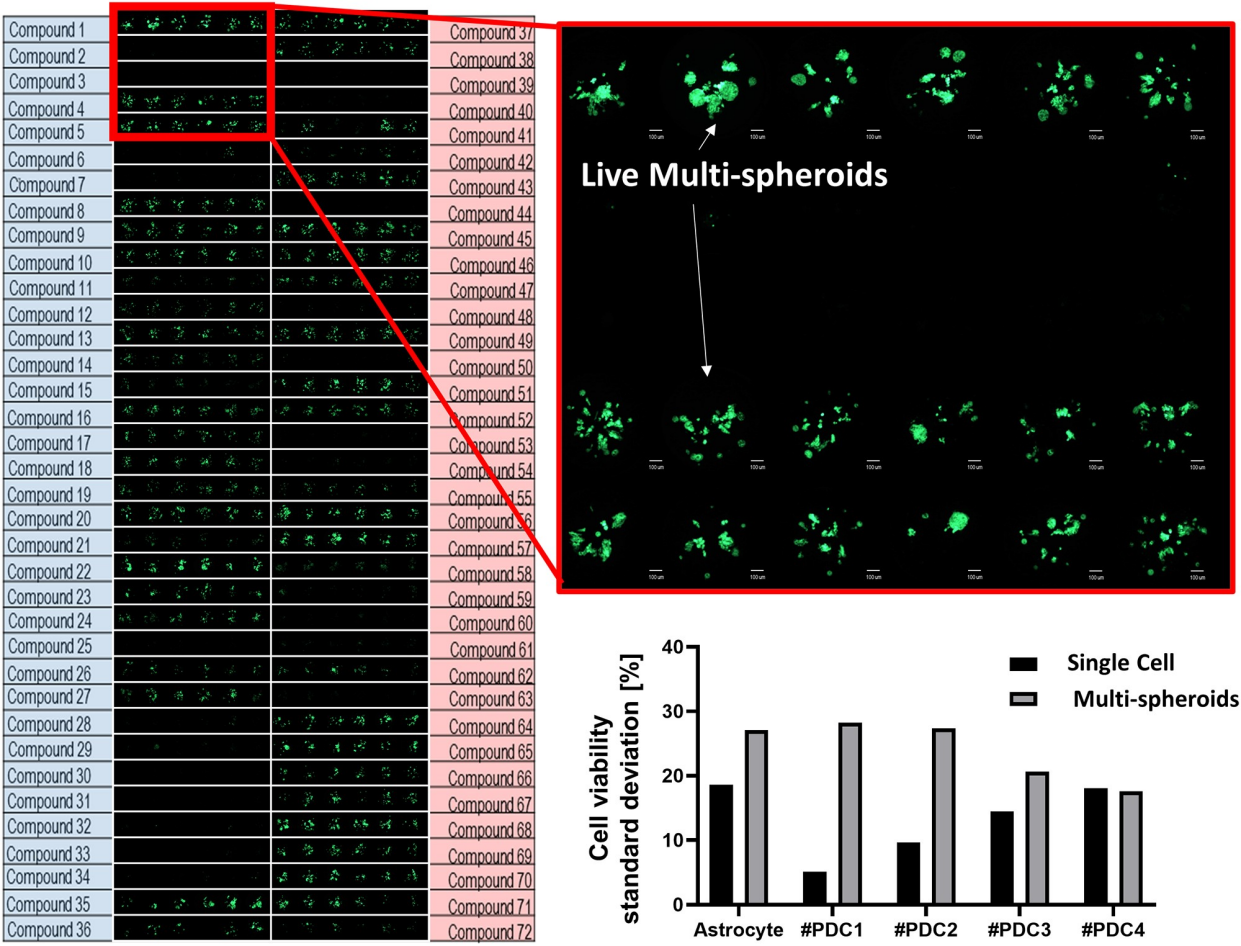
Scanning image of micropillar chip exposed to 72 compounds (including 2 DMSO control). The enlarged image shows stained live multi-spheroids (technical 6 replicates). The graph shows the standard deviation of cell viability in the DMSO control group in the single cell condition (7 day culture) and multi-spheroid condition (13 day culture).

Fig 3 shows the layout of 72 different compounds in the scanning image of the micropillar chip. Each compound has 6 replicates. The top row (compound 1, 37) is the DMSO control without compound treatment. The enlarged scanning images show live multi-spheroids (green lumps) treated with different compounds. An empty (block) circle means multi-spheroids were not viable due to effects of the compounds.

We compared the response of compounds to single cells and multi-spheroids separately. Single cells were treated with the different compounds on Day 1 and stained on Day 7. For multi-spheroids, compounds were added at Day 7 (after cells form multi-spheroids) and stained at Day 13, as shown in (Fig 2). In a typical drug screening, drugs are exposed for twice the cell doubling time. In the case of GBM patient-derived cells, the doubling time is approximately 3 to 4 days. Thus, we exposed the compound for 7 days.

### Viability measurement in single cell and multi-spheroid conditions

**Fig 4:**
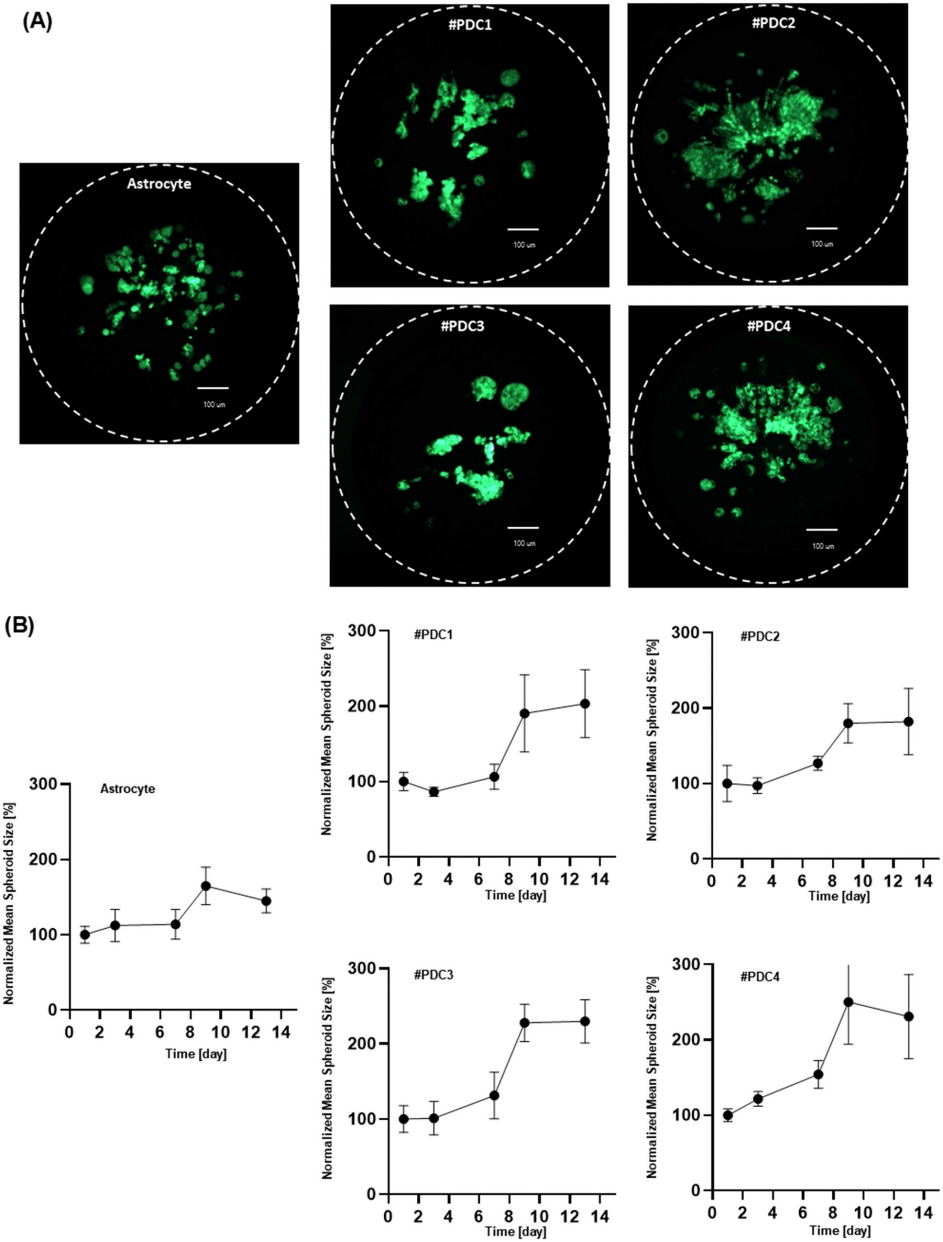
Multi-spheroid formation on a chip. (A) Live image of multi-spheroids of astrocytes (brain normal cell) and four GBM patient-derived cells after 13 days of culturing (B) Growth curves of 3D cultured cells on the chip.

Live multi-spheroids were stained using Calcein AM (4 mM stock from Invitrogen). The staining dye solution was prepared by adding 1.0 μL of Calcein AM in 8 mL staining buffer (MBD-STA50, Medical & Bio Device, South Korea). Cells were incubated with staining solution for 1 hour at 37°C in a 5% CO_2_-humidified atmosphere. One hour of staining time was sufficient to stain multi-spheroids. Multi-spheroids on the micropillar chip were scanned to measure viability quantitatively after staining the alginate spots. As shown in Fig 3, scanned images were obtained with an automatic optical fluorescence scanner (ASFA™ Scanner ST, Medical & Bio Device, South Korea). The total green-fluorescent intensity from live multi-spheroids in each spot was extracted using Chip Analyzer (Medical & Bio Device, South Korea). The software extracted total green- fluorescent intensities (8 bit green code among RGB code: 0~255) from living multi-spheroids in each cell spot on the scanned chip by setting up an analysis boundary (the circles in Fig 4). To resolve the background noise signal (Blurry green intensity spots which indicate low viable cells), only the green fluorescence representing the living cells was segmented and the fluorescence intensity value was extracted. The relative multi-spheroid viabilities were calculated by dividing green intensities of compounds with the average value of the DMSO controls (no compound treatment).

### Statistical analysis

**Fig 5:**
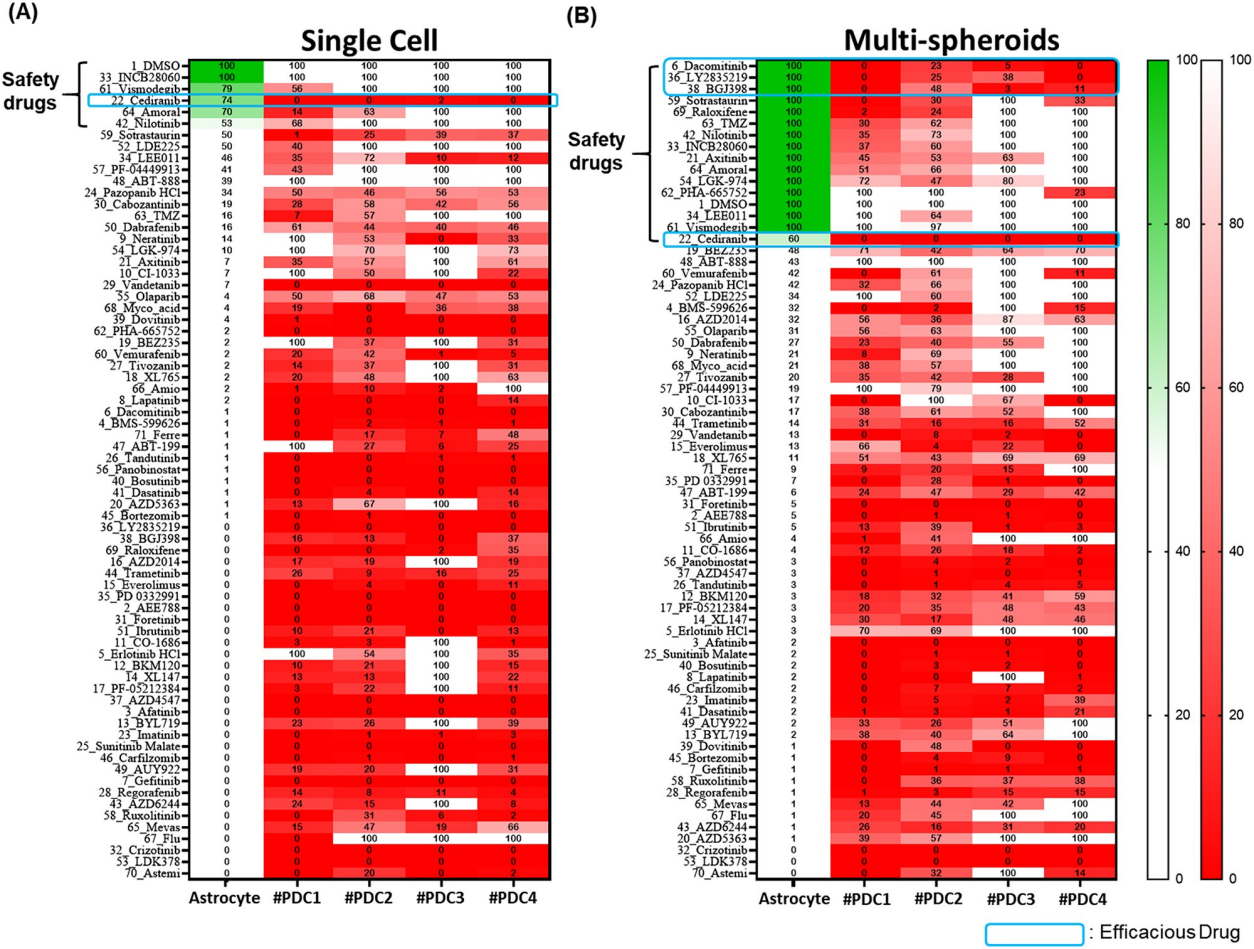
Relative cell viabilities when single cells or multi-spheroids were exposed to drugs for 7 days. Cell viability was measured using the green fluorescence intensity of living cells. The relative cell viabilities were normalized based on the cell viabilities of DMSO control. The safety drugs displayed low cytotoxicity due to more than 50% astrocyte viability. Efficacy drugs among safety drugs mean high efficacy due to less than 50% PDCs viability. Green color shading indicates no cytotoxicity and more than 50% astrocyte viability. (A) Single cell condition. (B) Multi-spheroids condition. Six different technical replicates (technical n = 6 per patient and normal cell) and fivedifferent biological replicates from PDCs and astrocyte (biological n = 4 patients vs. astrocytes) were included.

**Fig 6:**
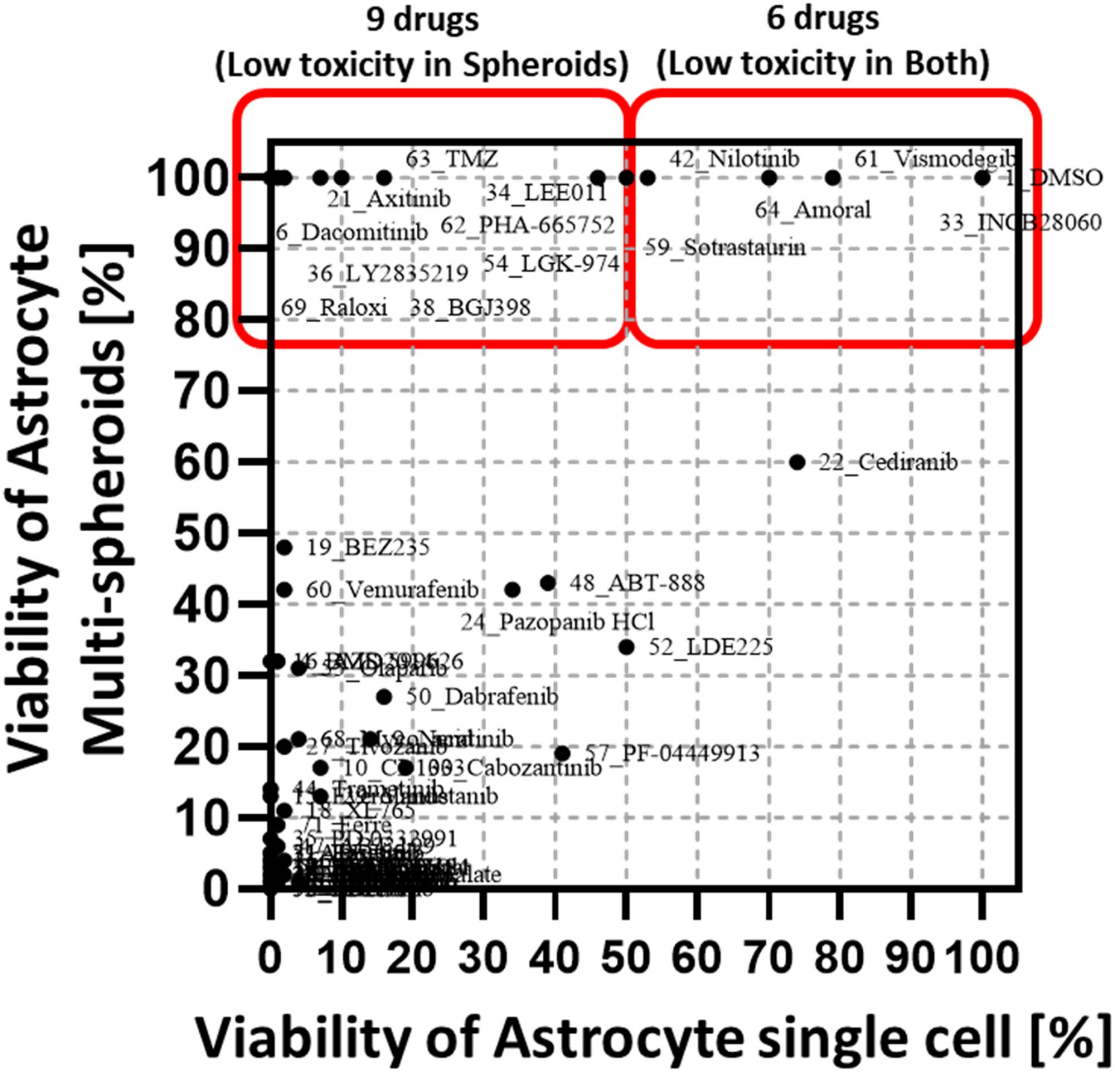
Comparison of cell viabilities of single cell and multi-spheroid clusters of astrocytes exposed to 70 drug compounds for 7 days. Six compounds show low toxicity in single cell and multi-spheroid conditions and nine drugs show low toxicity only in the multi-spheroid condition.

**Fig 7:**
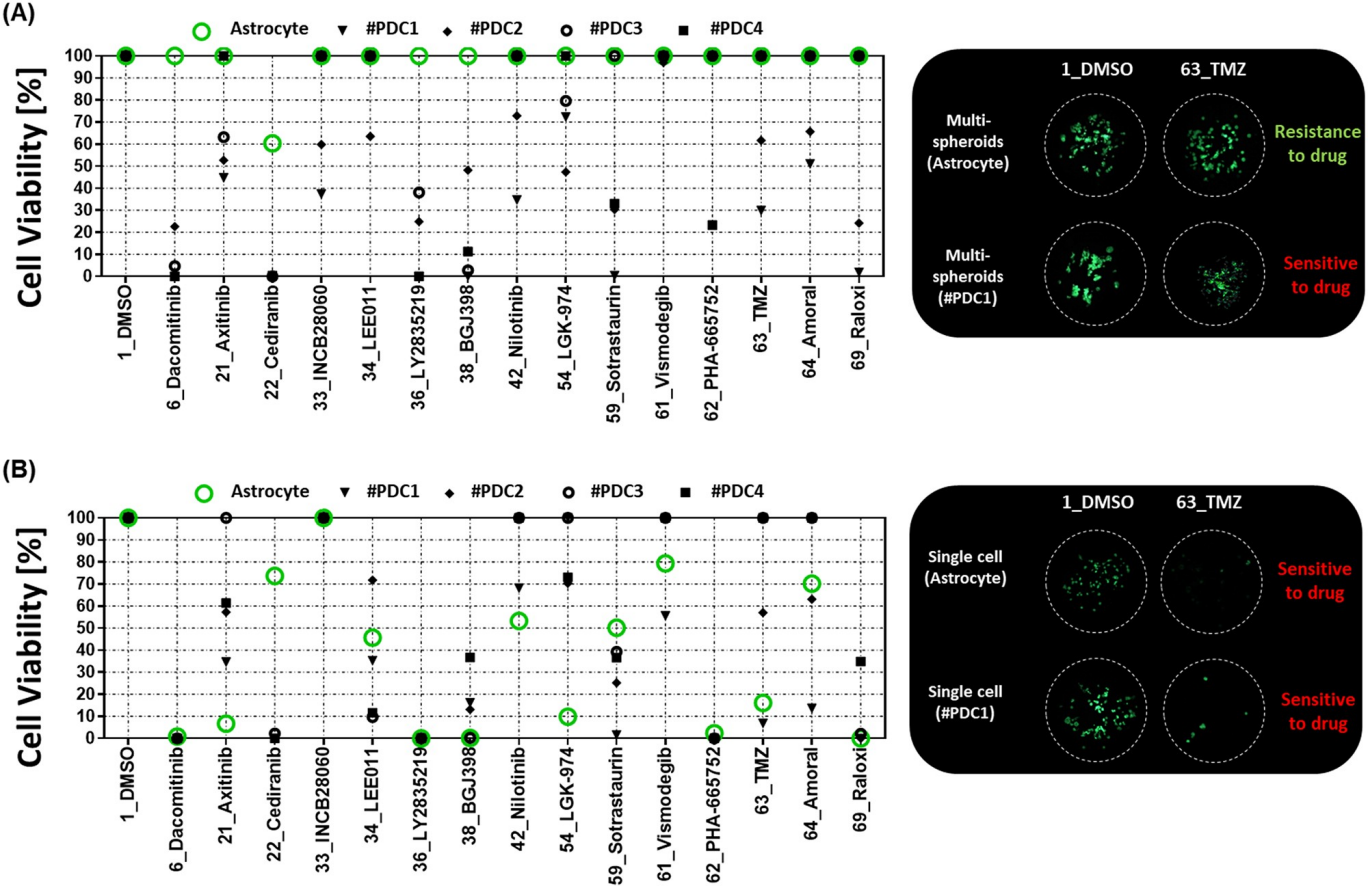
Compound heat map of multi-spheroid and single cell conditions. (A) In the multi-spheroids condition, cells were cultured for 7 days to form multi-spheroids (spheroid diameter over 100 μm) and multi-spheroids were treated with compounds for a further 7 days. (B) In the single cell condition, compounds were exposed to single cell 1 day after seeding cell and single cells were treated with compounds for 7 more days.

All data were expressed as the mean of six replicates. the size of multi-spheroids deviated approximately 30%. Patient-derived cells formed variable spheroid sizes, therefore p-values of the six replicates of each drug were calculated to identify drugs whose viability differences are statistically significant compared to controls (no drug condition). Efficacy values were compared by Student t-test using GraphPad Prism 9.0. Potency drugs were selected by comparing the viability of PDCs and viability of astrocytes. P-values < 0.05 were considered statistically significant. Drug efficacy was analyzed among the 6 different technical replicates (technical n = 6 per patient and normal cell) as well as 5 different biological replicates from PDCs and astrocytes (biological n = 4 patients vs. astrocytes) in Figs 3, 5–7.

### Ethical statement

This investigation was conducted in accordance with the ethical standards of the Declaration of Helsinki and national and international guidelines and was approved by the Institutional Review Board at Samsung Medical Center in Seoul, Korea. (IRB No. 201512092, IRB No. 201004004) Participant consents were confirmed in document form and minors did not include this study.

## Results and discussion

Previously, the efficacy and cytotoxicity of 70 different drug compounds were screened using a micropillar/microwell chip platform [18]. Compared to Temozolomide (TMZ), one of the most popular drugs for GBM, we found that cediranib showed high drug efficacy to GBM patient derived cells without toxicity. However, many compounds including TMZ showed cytotoxicity against normal glial cells (astrocytes) based on the drug response for single cells when the drug was added before the cells formed colonies. In the current paper, we modified a micropillar/microwell chip platform for long-term cell culture to form cancer multi-spheroids. Spacer gaps were introduced to allow CO_2_ exchange and incubation chambers were designed to prevent evaporation of media. With a conventional U-bottom culture method, it is difficult to carry out long-term cell cultures to form spheroids (S1A Fig). In order to carry out a long-term cell culture, the culture medium must be changed which poses a risk that the cells may be damaged or lost during this process. When using the U-bottom plate to culture the cells in a spheroid structure, the cells should not attach directly to the well because the cells need to move to the center of the U-bottom well by gravity. Thus, in general, extracellular matrix (ECM) is not used when culturing spheroids using U-bottom plates. For this reason, this culture method using U-bottom plates does not reflect the cell-ECM interactions in 3D cell culture, and also requires careful medium change to prevent loss of unattached cells. This creates a bottleneck for high-throughput screening and automation. However, the micropillar platform provides an advantage as the cells are mixed with the ECM reflecting cell-ECM interactions and fixed on the surface of the micropillar chip. Cells are simply transferred to a new microwell chip containing fresh media and drug compounds, avoiding the risk of cell damage (S1B Fig). As such, there is a considerable advantage when culturing cells for a long period of time. We used the array to grow cancer multi-spheroids for 7 days, after which the multi-spheroids were exposed to 70 compounds for another 7 days. Drug efficacy and cytotoxicity were measured. As shown in Fig 4 normal astrocytes and four GBM patient-derived cells formed mature spheroids (or multi-spheroids) whose size were more than 100 μm in diameter.

### Toxicity test (astrocytes) with multi-spheroids

As shown in (Fig 5), most compounds show high cytotoxicity when exposed to single cells for 7 days. Most compounds had similar cytotoxicity when used on either single cells or multi-spheroid astrocytes. However, fifteen drug compounds demonstrated low cytotoxicity to multi-spheroids (mature spheroid) or astrocytes (viability is higher than 50%). Of these 15 drug compounds, six showed low cytotoxicity for single cells while 9 compounds showed low cytotoxicity in both single cells and multi-spheroids, as shown in Fig 6. In previous research TMZ, which is widely known for its low cytotoxicity, exhibited high toxicity (with astrocyte viability < 16%) after treating the cells for 7 days but showed low toxicity (with astrocyte viability > 90%) after a 3-day treatment period. Previous studies assessed the cytotoxicity of TMZ using only a 3-day treatment period [24]. Astrocyte multi-spheroids, TMZ showed no cytotoxicity even though it was treated for 7-days. TMZ is commonly used to treat GBM cancer patients. Using TMZ drug testing results in multi-spheroids of astrocyte as a benchmark, more low cytotoxicity compounds were found such as Dacomitinib, Axitinib, LEE011, LY2835219, BGJ398, LGK-974, PHA-665752, Amoral and Raloxifene.

### Efficacy test of 70 drug compounds (four patient-derived GBM cells) in the multi-spheroid condition

The cell viabilities of normal astrocytes and four patient-derived GBM cells treated with different drug compounds, either in single cell or multi-spheroids conditions, are shown in Fig 5. Most of the targeted compounds exhibited high efficacy with 20 μM dosages in both single cell and multi-spheroid conditions. In previous work [25] when single cells were exposed to drug compounds for three days, cediranib exhibited high efficacy in all four patient-derived GBM cells and exhibited no cytotoxicity towards astrocytes. When treated for 7 days in the single cell condition, most compounds showed high cytotoxicity in astrocytes as well as high efficacy in four patient-derived GBM cells. Six compounds showed low cytotoxicity (viability>50%) but only cediranib and Sotrastaurin demonstrated high efficacy to four patient-derived GBM cells as shown in Fig 5. However, in multi-spheroids culture, fifteen compounds showed low cytotoxicity in astrocytes and four compounds (Dacomitinib, Cediranib, Ly2835219, BGJ398) showed high efficacy in four patient-derived cells (Fig 7).

Prior studies confirmed that more accurate drug response analysis is possible through volumetric analysis of 3D-cultured colonies in alginate spots [26]. Unfortunately, the volumetric analysis method is difficult to apply to micropillar/well chips as it requires image scanning using a confocal microscope. Additionally, the aim in the current paper was to measure drugs that showed specific efficacy only in tumor multi-spheroids compared to normal cells under the highest drug concentration condition. By testing this as a primary high-throughput drug screening concept, we were able to select efficacious drugs without volumetric analysis. Fig 3 shows the replication is six for each compound. The graph in Fig 3 shows the standard deviation of cell viabilities in single cell and multi-spheroids conditions and in no compound conditions (control). Single cell models showed under 20% standard deviation and the multi-spheroids model showed under 30% standard deviation. Big multi-spheroids showed higher variation. However, the most efficacious drugs (Dacomitinib, Cediranib, Ly2835219, BGJ398 selected in (Fig 7) had a P-value of less than 0.05. Compared to viability of no drug (control), multi-spheroid viabilities of four compounds were found to be statistically significant in GBM cells. Dacomitinib, Ly2835219, and BGJ398 showed high cytotoxicity when compounds were treated early in single cell culture before forming spheroids of astrocytes. However, mature spheroids (multi-spheroids) of astrocytes displayed high resistance to those three compounds while four patient-derived GBM cells had markedly reduced viability to those three compounds. In other research papers, those three compounds showed the high efficacy to GMM cells or patients. Abemaciclib (LY2835219), a drug that inhibits Cyclin-Dependent Kinases 4/6 and crosses the Blood-Brain Barrier, demonstrates *in vivo* activity against intracranial human brain tumor xenografts [26]. In preclinical tests, dacomitinib showed its effectiveness against glioblastoma [27]. BGJ398 is currently in Phase II clinical trials to assess anti-tumor efficacy in recurrent GBM patients (*NCT number*: NCT01975701). Our study showed that by employing our high-throughput multi-spheroids array model, we identified new drug candidates such as Dacomitinib, Ly2835219, BGJ398, and cediranib for GBM. This serves as a proof-of-concept study to identify new drug candidates forcancers using our high-throughput multi-spheroids array chip.

## Conclusion

A cancer multi-spheroid array chip was developed to be used for drug screening using micropillar and microwell structures. Cells in alginate were encapsulated in the chip and patient-derived cells were grown for more than 7 days to form cancer multi-spheroids. Importantly, this method also prevents accidental damage to multi-spheroids during cell maintenance and culturing. We used cells derived from patients with GBM, the most common and lethal form of central nervous system cancer, to validate the multi-spheroids array chip performance. After forming more than 100 μm-diameter multi-spheroids in 12 × 36 pillar array chip (25 mm × 75 mm), we treated the multi-spheroids with 70 different drug compounds (6 replicates each) to evaluate drug compound efficacy. We identified new drug candidates with high safety and efficacy for GBM using a high-dose drug heat map array.

## Supporting information

S1 Fig Comparison between the conventional U-bottom method and the micropillar/well chip. (TIF)

S1 Table Mean and standard deviation (SD) of cell viability (n = 6). * P value (compared with DMSO) >0.05.
(PPTX)
