# Correction: High-dose drug heat map analysis for drug safety and efficacy in multi-spheroid brain normal cells and GBM patient-derived cells

**DOI:** 10.1371/journal.pone.0295827

**Published:** 2023-12-07

**Authors:** Sang-Yun Lee, Yvonne Teng, Miseol Son, Bosung Ku, Ho Sang Moon, Vinay Tergaonkar, Pierce Kah-Hoe Chow, Dong Woo Lee, Do-Hyun Nam

An additional affiliation is missing for the eighth author. Dong Woo Lee is also affiliated with Central R & D Center, Medical & Bio Device (MBD) Co., Ltd, Suwon, 16229, Republic of Korea.
