# Supplementary figures and images for: High-dose drug heat map analysis for drug safety and efficacy in multi-spheroid brain normal cells and GBM patient-derived cells
Source: PLoS One. 2021 Dec 2;16(12):e0251998. doi: 10.1371/journal.pone.0251998 (PMC8638871; doi:10.1371/journal.pone.0251998)

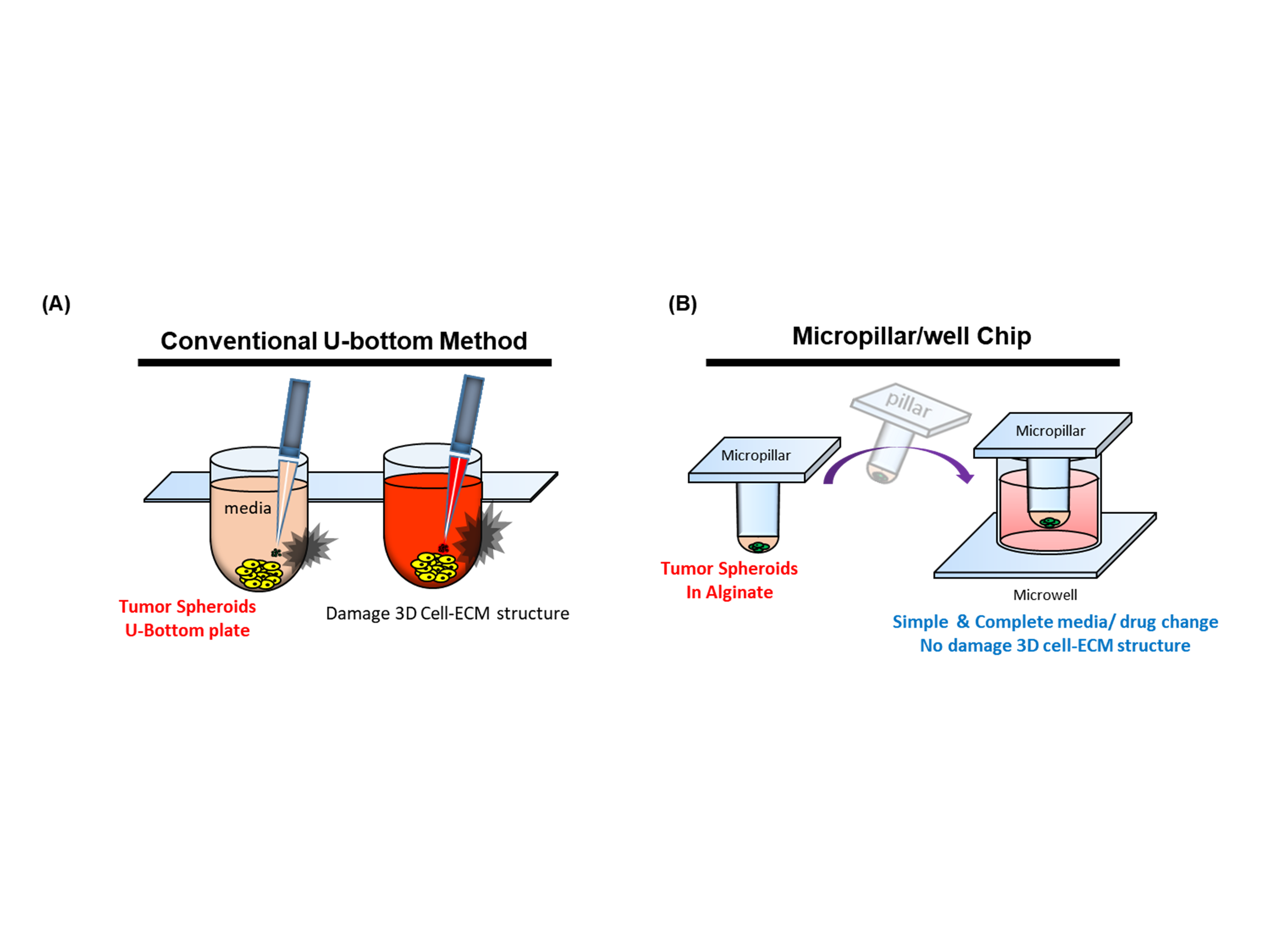

Supplement: S1 Fig — (TIF) [file pone.0251998.s001.tif]
