# Supplementary material for: High-dose drug heat map analysis for drug safety and efficacy in multi-spheroid brain normal cells and GBM patient-derived cells
Source: PLoS One. 2021 Dec 2;16(12):e0251998. doi: 10.1371/journal.pone.0251998 (PMC8638871; doi:10.1371/journal.pone.0251998)
Supplement: S1 Table — * P value (compared with DMSO) >0.05. (PPTX) [file pone.0251998.s002.pptx]

## Slide 1
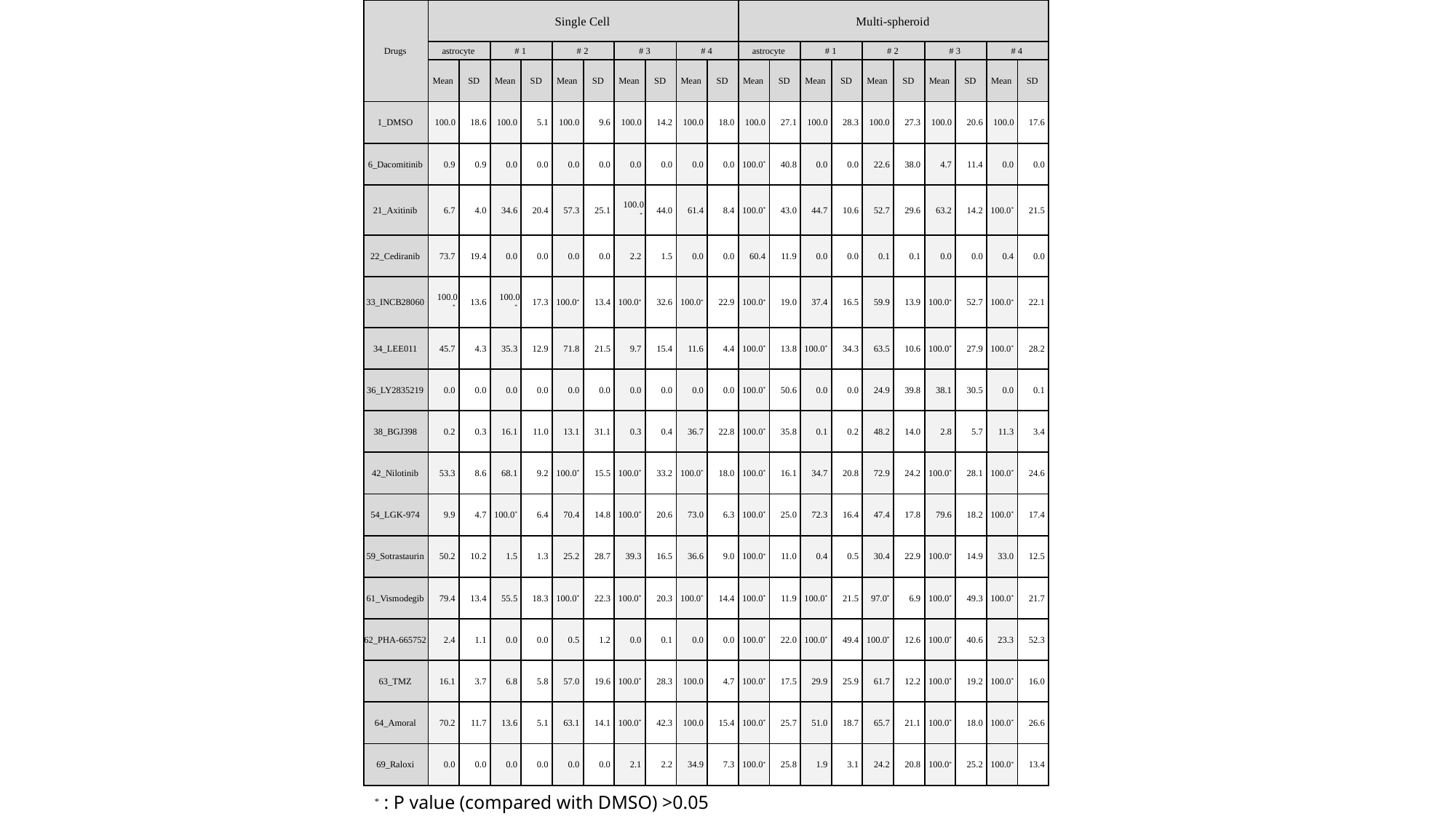

| Drugs | Single Cell | | | | | | | | | | Multi-spheroid | | | | | | | | | |
| --- | --- | --- | --- | --- | --- | --- | --- | --- | --- | --- | --- | --- | --- | --- | --- | --- | --- | --- | --- | --- |
| | astrocyte | | # 1 | | # 2 | | # 3 | | # 4 | | astrocyte | | # 1 | | # 2 | | # 3 | | # 4 | |
| | Mean | SD | Mean | SD | Mean | SD | Mean | SD | Mean | SD | Mean | SD | Mean | SD | Mean | SD | Mean | SD | Mean | SD |
| 1\_DMSO | 100.0 | 18.6 | 100.0 | 5.1 | 100.0 | 9.6 | 100.0 | 14.2 | 100.0 | 18.0 | 100.0 | 27.1 | 100.0 | 28.3 | 100.0 | 27.3 | 100.0 | 20.6 | 100.0 | 17.6 |
| 6\_Dacomitinib | 0.9 | 0.9 | 0.0 | 0.0 | 0.0 | 0.0 | 0.0 | 0.0 | 0.0 | 0.0 | 100.0\* | 40.8 | 0.0 | 0.0 | 22.6 | 38.0 | 4.7 | 11.4 | 0.0 | 0.0 |
| 21\_Axitinib | 6.7 | 4.0 | 34.6 | 20.4 | 57.3 | 25.1 | 100.0 \* | 44.0 | 61.4 | 8.4 | 100.0\* | 43.0 | 44.7 | 10.6 | 52.7 | 29.6 | 63.2 | 14.2 | 100.0\* | 21.5 |
| 22\_Cediranib | 73.7 | 19.4 | 0.0 | 0.0 | 0.0 | 0.0 | 2.2 | 1.5 | 0.0 | 0.0 | 60.4 | 11.9 | 0.0 | 0.0 | 0.1 | 0.1 | 0.0 | 0.0 | 0.4 | 0.0 |
| 33\_INCB28060 | 100.0 \* | 13.6 | 100.0 \* | 17.3 | 100.0\* | 13.4 | 100.0\* | 32.6 | 100.0\* | 22.9 | 100.0\* | 19.0 | 37.4 | 16.5 | 59.9 | 13.9 | 100.0\* | 52.7 | 100.0\* | 22.1 |
| 34\_LEE011 | 45.7 | 4.3 | 35.3 | 12.9 | 71.8 | 21.5 | 9.7 | 15.4 | 11.6 | 4.4 | 100.0\* | 13.8 | 100.0\* | 34.3 | 63.5 | 10.6 | 100.0\* | 27.9 | 100.0\* | 28.2 |
| 36\_LY2835219 | 0.0 | 0.0 | 0.0 | 0.0 | 0.0 | 0.0 | 0.0 | 0.0 | 0.0 | 0.0 | 100.0\* | 50.6 | 0.0 | 0.0 | 24.9 | 39.8 | 38.1 | 30.5 | 0.0 | 0.1 |
| 38\_BGJ398 | 0.2 | 0.3 | 16.1 | 11.0 | 13.1 | 31.1 | 0.3 | 0.4 | 36.7 | 22.8 | 100.0\* | 35.8 | 0.1 | 0.2 | 48.2 | 14.0 | 2.8 | 5.7 | 11.3 | 3.4 |
| 42\_Nilotinib | 53.3 | 8.6 | 68.1 | 9.2 | 100.0\* | 15.5 | 100.0\* | 33.2 | 100.0\* | 18.0 | 100.0\* | 16.1 | 34.7 | 20.8 | 72.9 | 24.2 | 100.0\* | 28.1 | 100.0\* | 24.6 |
| 54\_LGK-974 | 9.9 | 4.7 | 100.0\* | 6.4 | 70.4 | 14.8 | 100.0\* | 20.6 | 73.0 | 6.3 | 100.0\* | 25.0 | 72.3 | 16.4 | 47.4 | 17.8 | 79.6 | 18.2 | 100.0\* | 17.4 |
| 59\_Sotrastaurin | 50.2 | 10.2 | 1.5 | 1.3 | 25.2 | 28.7 | 39.3 | 16.5 | 36.6 | 9.0 | 100.0\* | 11.0 | 0.4 | 0.5 | 30.4 | 22.9 | 100.0\* | 14.9 | 33.0 | 12.5 |
| 61\_Vismodegib | 79.4 | 13.4 | 55.5 | 18.3 | 100.0\* | 22.3 | 100.0\* | 20.3 | 100.0\* | 14.4 | 100.0\* | 11.9 | 100.0\* | 21.5 | 97.0\* | 6.9 | 100.0\* | 49.3 | 100.0\* | 21.7 |
| 62\_PHA-665752 | 2.4 | 1.1 | 0.0 | 0.0 | 0.5 | 1.2 | 0.0 | 0.1 | 0.0 | 0.0 | 100.0\* | 22.0 | 100.0\* | 49.4 | 100.0\* | 12.6 | 100.0\* | 40.6 | 23.3 | 52.3 |
| 63\_TMZ | 16.1 | 3.7 | 6.8 | 5.8 | 57.0 | 19.6 | 100.0\* | 28.3 | 100.0 | 4.7 | 100.0\* | 17.5 | 29.9 | 25.9 | 61.7 | 12.2 | 100.0\* | 19.2 | 100.0\* | 16.0 |
| 64\_Amoral | 70.2 | 11.7 | 13.6 | 5.1 | 63.1 | 14.1 | 100.0\* | 42.3 | 100.0 | 15.4 | 100.0\* | 25.7 | 51.0 | 18.7 | 65.7 | 21.1 | 100.0\* | 18.0 | 100.0\* | 26.6 |
| 69\_Raloxi | 0.0 | 0.0 | 0.0 | 0.0 | 0.0 | 0.0 | 2.1 | 2.2 | 34.9 | 7.3 | 100.0\* | 25.8 | 1.9 | 3.1 | 24.2 | 20.8 | 100.0\* | 25.2 | 100.0\* | 13.4 |
* : P value (compared with DMSO) >0.05
